# Differences in Mortality Rates Between Services Users of Different Racial/ethnic Groups

**DOI:** 10.1192/bjo.2026.11582

**Published:** 2026-06-30

**Authors:** Filipa M.A.A. Teixeira, Motaz Sonbol, Mohamed Anis-Alavi

**Affiliations:** 1 South West London and St. George's Mental Health NHS Trust, London, United Kingdom; 2 Guy's and St Thomas NHS Foundation Trust, London, United Kingdom; 3 Kings College London, London, United Kingdom

## Abstract

**Aims:**

The purpose of this thematic review was to analyse and explore the underlying factors contributing to differences in mortality rates among service users from different racial and ethnic groups within South West London and St George’s Mental Health NHS Trust (SWLStG), specifically in ethnic minority groups. By systematically examining deaths occurring under the care of the Trust, the review aimed to identify recurring themes, potentially to inform recommendations to reduce inequalities and improve patient safety.

**Methods:**

Information was collected on all reported deaths between February 2025 and April 2025 of service users under the care of the Trust’s mental health services. A total of 145 deaths were reported and included in the review. Incident reports were extracted from Ulysses. Demographic and clinical information was checked and verified using RIO, including recorded ethnicity, progress notes, and relevant clinical and risk assessment documentation.

**Results:**

Of the 145 deaths reviewed, most occurred among White British (66%) or “other white” service users (10%), and of confirmed or suspected suicides, 73% were from white British or “other white” service users. 6.21% of service users’ ethnicity was unknown. Most deaths (84%) were due to natural causes, often at relatively older ages and in the context of significant physical health comorbidity. A proportion of deaths occurred following loss of contact with services, due to patient disengagement or during transition between mental health teams. There was limited information available for some deaths, making cause and circumstance unclear. Variation in service engagement and clinical documentation was observed across teams. Most service users had a documented mental disorder, most commonly cognitive decline or dementia (27%) and mixed anxiety and depression (25%). Among patients who died by possible suicide, 31.8% had a history of drug or alcohol dependence. No clear ethnicity-specific themes were identified, with interpretation limited by the small sample size.

**Conclusion:**

This review highlights that most deaths among mental health service users were due to natural causes and were associated with physical health comorbidities. Recurrent themes included disengagement from services, communication gaps during care transitions, and variable documentation. Although ethnic minorities were a small proportion of the sample, evidence of lower access of these populations to mental health services remains relevant. Improving consistency of ethnicity recording, clinical documentation, engagement with service users, as well as strengthening communication between services, is indicated.

